# Cytokine profiles amongst Sudanese patients with visceral leishmaniasis and malaria co-infections

**DOI:** 10.1186/1471-2172-15-16

**Published:** 2014-05-01

**Authors:** Erika van den Bogaart, Al-Badawi A Talha, Masja Straetemans, Pètra F Mens, Emily R Adams, Martin P Grobusch, Bakri Y M Nour, Henk D F H Schallig

**Affiliations:** 1 Department of Biomedical Research, Royal Tropical Institute (KIT), Amsterdam, The Netherlands; 2 Department of Medical Parasitology, Faculty of Medical Laboratory Sciences, University of Gezira, Wad Medani, Sudan; 3 Department of Infectious Diseases, Division of Internal Medicine, Center of Tropical Medicine and Travel Medicine, Academic Medical Center, University of Amsterdam, Amsterdam, The Netherlands; 4 Department of Medical Parasitology, Blue Nile National Institute for Communicable Diseases, University of Gezira, Wad Medani, Sudan; 5 Parasitology Department, Liverpool School of Tropical Medicine, Liverpool, United Kingdom

**Keywords:** Visceral leishmaniasis, Malaria, Co-infection, Cytokines, Sudan

## Abstract

**Background:**

The immune system plays a critical role in the development of co-infections, promoting or preventing establishment of multiple infections and shaping the outcome of pathogen-host interactions. Its ability to mediate the interplay between visceral leishmaniasis (VL) and malaria has been suggested, but poorly documented. The present study investigated whether concomitant infection with *Leishmania donovani* complex and *Plasmodium falciparum* in naturally co-infected patients altered the immunological response elicited by the two pathogens individually.

**Results:**

Circulating levels of interferon (IFN)-γ, interleukin (IL)-2, IL-4, IL-6, IL-10, IL-12p70, IL-13, IL-17A and tumor necrosis factor (TNF) were assessed in sera of patients infected with active VL and/or malaria and healthy individuals from Gedarif State, Sudan. Comparative analysis of cytokine profiles from co- and mono-infected patients highlighted significant differences in the immune response mounted upon co-infection, confirming the ability of *L. donovani* and *P. falciparum* to mutually interact at the immunological level. Progressive polarization towards type-1 and pro-inflammatory cytokine patterns characterized the co-infected patients, whose response partly reflected the effect elicited by VL (IFN-γ, TNF) and malaria (IL-2, IL-13), and partly resulted from a synergistic interaction of the two diseases upon each other (IL-17A). Significantly reduced levels of *P. falciparum* parasitaemia (*P* <0.01) were detected in the co-infected group as opposed to the malaria-only patients, suggesting either a protective or a non-detrimental effect of the co-infection against *P. falciparum* infection.

**Conclusions:**

These findings suggest that a new immunological scenario may occur when *L. donovani* and *P. falciparum* co-infect the same patient, with potential implications on the course and resolution of these diseases.

## Background

Co-infection of a host by multiple parasite species is a commonly observed condition, where individual susceptibility and infectiousness are affected at various levels [1-3]. Inter-microbial competition along with micro-environmental and immunological conditioning govern the magnitude and type of interactions across poly-parasitism, promoting or hampering establishment of multiple parasite infections and their ability to persist and spread to new patients [4]. At the immunological level, cross-regulation of pathogen-associated pathways is achieved through cytokine signaling; an integrated network responsible for the controlled polarization and amplification of immune responses [5,6]. As a result, cytokines secreted in response to one parasite species may act synergistically or antagonistically with those elicited by another species, enhancing (cross-immunity) or impairing (immune-suppression) simultaneous control of infections. Cytokine ability to shape the immune system into efficacious responses arises from their downstream actions on the effector mechanisms, with great variation across different host-parasite systems [6,7]. Conversely, upstream cytokine functions are more stereotypical, while remaining predictive of effector efficacy. Hence, their characterization in poly-parasitized models provide a valuable and convenient framework for understanding co-infection dynamics [6,8].

Visceral leishmaniasis (VL) and malaria are two major parasitic diseases which overlap geographically and may co-exist in the same patients [9,10]. Partially sharing the same host tissue niches, the two infections have the ability of evading and subverting immune surveillance, with clinical outcomes largely dependent on the immunological status of the host. Obligate intracellular parasites of the visceralizing *Leishmania donovani* complex successfully colonize macrophages and other reticulo-endothelial cells of the lymphoid system, by altering signaling pathways associated with parasite killing and adaptive immunity engagement [11,12]. As a result, phagocytes harboring *Leishmania* parasites are incapacitated to function as cytolytic and T-cell priming effectors, causing immune dysfunction and tissue injury. Resistance to infection is conferred by development of effective T helper cell 1-type (Th1) responses, mounted upon release of a pleiotropic interleukin (IL)-12 and interferon (IFN)-γ cytokine network, and boosted by pro-inflammatory (tumor necrosis factor (TNF), IL-23, IL-17A) and Th2-promoting (IL-4) mediators [13-17]. Thus, in contrast to the classical Th1-Th2 paradigm suiting predictions of resistance/susceptibility to cutaneous leishmaniasis [12], clearance of *L. donovani* appears to be blunted by induction of the regulatory T cell subset Tr1, rather than Th2 or Th3 clusters, through an IL-10 mediated mechanism [18-20]. Anergic IL-10 producing T cells have also been detected in response to *Plasmodium falciparum* infections [21-25], which account for the largest proportion of malaria disease. Complex, stage-specific networks of antibody-dependent and cell-mediated interactions provide immunity against *Plasmodium* spp., with clinical implications depending on the type and timing of cytokine release. Early type-1 responses, dominated by IFN-γ, IL-2 and TNF, have been reportedly associated with inhibition of liver stage development [26-31], resolution of acute malaria parasitaemias [32-34] and delay of re-infection [35], as confirmed by the absolute requirement of IFN-γ in the effector mechanism of sporozoite-induced protective immunity [35-38]. Release of these cytokines, initiated by the innate immune system (Natural killer (NK) cells, γδT- and αβT-cells) [39-41] and sustained by *Plasmodium*-specific CD8^+^ and CD4^+^ cells [32,37,39], requires to be timely counterbalanced by a switch to type-2 responses which propagate humoral immunity against the erythrocytic stage, and limit the pathogenicity of pro-inflammatory cytokines [42,43]. Similar symptom-suppressing activities appear to be mediated by IL-10 and transforming growth factor (TGF)-β, which in the attempt to reduce immunopathology, can interfere with the protective effects of IFN-γ and TNF and allow the parasite to grow uncontrollably [21,44,45].

Despite VL and malaria co-infection cases being encountered across co-endemic areas, little work has been done so far to examine the dynamics of this co-infection and its effect on host immunity. Studies performed in co-infection murine models of *P. chabaudi chabaudi* and *L. infantum*[46], and of *P. yoelii* and *L. mexicana amazonensis*[47,48] have highlighted an exacerbating effect of the two diseases upon each other, particularly for leishmaniasis, whose enhanced parasite load was attributed to the *Plasmodium*-triggered release of splenic IL-4, as assessed by gene expression [46]. Conversely in golden hamsters, pre-inoculation with different *L. infantum* strains resulted in a reduced proliferation of *P. berghei*, with no aggravation of the *Leishmania* infection [49]. Whilst these discrepancies reflect the difficulty in extrapolating animal model data, particularly when dealing with multiple infections, they agree on recognizing the immune system as a major determinant of *Leishmania* and *Plasmodium* spp. interactions upon co-infection.

In the present study, the cytokine profiles of naturally co-infected patients were examined. Blood samples from patients actively infected with VL and/or malaria and from healthy individuals were collected during an exploratory survey conducted in Gedarif State, Sudan, and the level of nine different cytokines selected from across the four major response arms of the immune system were assessed simultaneously. The comparative analysis between co- and mono-infected groups highlighted substantial differences in the cytokine profile of these patients and their levels of *P. falciparum* parasitaemia, emphasizing the importance of immune-mediated interactions in poly-parasitism.

## Methods

### Study site, study cases and ethical considerations

The sample collection was performed in February 2011 in the village of Tabarak Allah, an endemic area of *L. donovani*, located in Gedarif State, Sudan. Patients were recruited at Tabarak Allah Hospital, which hosts a VL treatment center managed by Médecins sans Frontières since January 2010. Seasonal and unstable malaria prevails in the area, where co-infection rates of 18% to 45% were recorded amongst Tabarak Allah VL in-patients (2005-2010) [10].

All individuals included in the study originated from Gedarif State and aged six years or above. Eligibility for the study was precluded to children up to age six, due to immature status of their immune system [50,51]. Individuals with previous history of VL were also excluded to ensure relapse cases were not enrolled in the study. Included patients reported no history of immune-related disorders, or of ongoing infectious diseases (other than VL and malaria). Clinical and laboratory examinations were performed, including assessment of hemoglobin levels (by HemoCue) and white blood cell (WBC) counts (by microscopy), and their outcomes recorded on anonymized case record forms. Plasma and serum were obtained from peripheral blood and stored at -70°C until tested. None of the subjects received anti-leishmanial chemotherapy before collection of blood samples, while a minimum of two-week lapse from previous treatment was observed for anti-malarial drugs. Written informed consent was obtained from each study participant above 18 years of age or guardian who consented on their behalf, after providing information on the study aim and procedures in the local language. The survey was conducted with the approval of the Sudanese Minister of Health (National Research Ethics Review Committee), who granted National Ethical Clearance (Nr. 140-10-11).

### Diagnostic algorithm

For categorization of study subjects, the following diagnostic algorithm was implemented. All patients presenting at the study hospital with symptoms of VL and/or malaria, including fever, weight loss, hepato-splenomegaly and anemia, were given physical examination. Finger-prick blood was assessed by microscopy for diagnosis of malaria and by direct agglutination test (DAT) for diagnosis of VL. Assessment of *P. falciparum* parasitaemia was performed by microscopy, counting the total number of parasites per 200 WBCs, as previously described [52]. Artemisinin-based combination therapies were administered to patients positively diagnosed for malaria. The DAT was performed on filter paper-spotted blood, using freeze-dried antigen and control sera from the Royal Tropical Institute (Amsterdam, the Netherlands). A cut-off titer of 3,200 was used, as previously established for the area [53]. Accordingly, patients meeting the WHO clinical definition for VL (fever for >2 weeks with either anemia or splenomegaly) [54], and having a DAT titer >3,200, but no history of VL were diagnosed with primary active VL and received a 30-day course of parenteral sodium stibogluconate, conforming to the national policy, along with the anti-malarial regimen, if required. Patients who tested negative for both VL and malaria were excluded from the study and referred to the hospital medical staff for alternative diagnoses. In total, 102 participants (77 VL and/or malaria confirmed patients and 25 healthy controls) were included at study entry. Prior to initiation of specific chemotherapies, peripheral blood was collected from enrolled participants and processed to obtain serum and plasma samples. A second subsequent evaluation of all specimens to confirm (or exclude in case of healthy controls) diagnosis of VL and/or malaria was independently performed at the Royal Tropical Institute, the Netherlands. Specific antibodies to *Leishmania* were measured in sera or, when unavailable, filter paper-spotted blood using the DAT and two commercially available rk39 tests, the DiaMed IT-Leish® (Diamed AG, Cressier sur Morat, Switzerland) and the Kalazar Detect™ (InBios International, Inc., Washington, USA). The following conditions were considered indicative of VL infection: a) DAT titers ≥3,200, with or without positive rk39 test outcomes; b) DAT titers =1,600 with at least one confirmatory rk39 test; c) DAT titers <1,600 with positive result in the field (>3,200) and a positive rk39 test. Samples that did not fulfill these criteria were excluded from the study (*n* = 8) or re-categorized (*n* = 3), if tested positive for malaria only. Thin and thick blood smears of all study participants were microscopically re-assessed to confirm or exclude presence of *P. falciparum* parasites. When slide re-examination resulted in discordant outcomes, a rapid test (SD Bioline, Standard Diagnostics, Inc., Korea) for detection of *P. falciparum* and *Plasmodium* spp. was performed on the corresponding serum sample. Positive results obtained with the serological test were considered confirmatory of malaria cases (*n* = 4), while specimens which tested negative were excluded from the analysis (*n* = 2). In addition, five other cases (3 healthy controls, 1 co-infected patient and 1 malaria patient) were excluded from the study, due to poorly reliable test outcomes, missing samples or diagnosis of non-*P. falciparum* malaria. From the 102 participants included at study entry, 15 were excluded because they did not match the diagnostic criteria, narrowing the sample size to 87 cases.

### Clinical groups

#### Group 1

Primary VL cases (*n* = 29), defined as VL-seropositive individuals who fulfilled the clinical case definition of VL and tested negative for malaria.

#### Group 2

Clinical malaria patients (*n* = 21). This group included parasitologically-confirmed cases of *P. falciparum* malaria who presented at hospital with clinical symptoms, such as fever, hepato-splenomegaly and anemia, and lacked *Leishmania*-specific antibodies.

#### Group 3

VL and malaria co-infected patients (*n* = 15), defined as VL-seropositive individuals diagnosed with a *P. falciparum* malaria infection.

#### Group 4

Healthy endemic controls (*n* = 22) with a VL-seronegative profile and no microscopically detectable malaria in peripheral blood.

### Cytokine measurement

Cytokine levels in patients’ samples were determined using a 9-milliplex magnetic bead-based immunoassay (HCYTOMAG-60 K, Millipore BV, Amsterdam, the Netherlands), performed according to manufacturer’s instructions. Briefly, 25 μL of magnetic beads internally labeled with multiple fluorophores and coated with specific capture antibodies against one of the nine cytokines (TNF, IL-2, IL-4, IL-6, IL-10, IL-12p70, IL-13, IL-17A and IFN-γ) was added to 25 μL of patient sample and an equal amount of assay buffer. Standards and quality controls for each cytokine were mixed likewise. After an overnight incubation followed by extensive wash to remove unbound proteins, 25 μL of biotinylated detection antibodies was added and the fluorescence of the streptavidin-phycoerythrin complex measured by a MAGPIX® (Luminex, Austin, USA). A minimum of 50 beads per cytokine was measured. Interpolation of sample concentrations using a five-parameter logistic standard curve was performed with the MILLIPLEX® Analyst 5.1 software (Merck Millipore, Billerica, USA). The lower detection limits of the assay were: 0.53 pg/mL for TNF, 0.54 pg/mL for IL-2, 0.34 pg/mL for IL-4, 0.97 pg/mL for IL-6, 0.59 pg/mL for IL-10, 0.74 pg/mL for IL-12p70, 0.57 pg/mL for IL-13, 0.22 pg/mL for IL-17A and 0.17 pg/mL for IFN-γ. Comparative analysis of cytokine profiles included both serum (*n* = 83) and, when unavailable, plasma samples (*n* = 5), since exclusion of plasma assessments had no effect on the outcome of the analysis.

### Statistical analysis

Group-wise comparison of cytokine values was performed using nonparametric statistics. Mann-Whitney U test and Kruskal-Wallis test were used to examine whether continuous variables from two or multiple groups, respectively, originated from the same distribution, whilst comparison of categorical variables was performed using the Chi-square test. *P* values <0.05 were considered indicative of statistical significance. Spearman’s (r_s_) rank correlations were computed to assess statistical dependence between cytokine levels and the corresponding patient demographic/clinical characteristics and between each cytokine pair. Data analysis was conducted with STATA software (College Station, TX, USA).

## Results

### Study population

The sex, age, hemoglobin level, WBC count and *P. falciparum* infection intensity of patients diagnosed with VL and/or malaria are summarized in Table 1. With the exception of malaria parasitaemia, which appeared significantly reduced amongst co-infected patients (*P* <0.01), no major differences in the baseline distribution of these variables were observed, with patients sharing most of their demographic and clinical characteristics. Mild anemia with normal leukocyte counts characterized most of the actively infected population, who largely consisted of young boys. Co-infected patients displayed some intermediate features between the VL and the malaria groups, including lower male to female ratio, younger age and milder anemia as compared with the VL patients.

**Table 1:** **Baseline characteristics of patients with VL and**/**or malaria recruited at Tabarak Allah Hospital**, **Sudan**

**Characteristics**	**Patient group**				***P *** **value**
	**1**	**2**	**3**	**Total**	
**Subjects ( *n *)**	29	21	15	65	
**Male/Female ( *n *)**	20/8^*a*^	11/9^*b*^	9/6	40/23^*c*^	0.5
**Age (years)**	19 (9-29)^*d*^	8 (7-26)	16 (9-21)	15 (8-24)^*e*^	0.2
**Hb level (g/dL)**	9.6 (7.1-12.4)	10.3 (9.1-11.2)	9.9 (7.5-11.5)	10.0 (8.4-11.4)	0.8
**WBC count/mm**^**3**^	5650 (4925-6900)^*f*^	6800 (5900-8000)^*g*^	5400 (4250-7150)^*h*^	6050 (4950-7400)^*i*^	0.2
**DAT titer**	25600 (6400-102400)	NA	12800 (6400-102400)	19200 (6400-102400)	0.9^†^
***P.f*. parasitaemia (parasites/μL)**	NA	825 (355-2325)^*j*^	53 (38-585)^*k*^	370 (51-1113)^*l*^	0.005^‡^

When not otherwise indicated, data shown represent median and (interquartile range).

*Hb* = hemoglobin; *WBC* = white blood cell; *DAT* = direct agglutination test; *P.f*. = *Plasmodium falciparum*; *NA* = not applicable.

Groups 1 to 3 are: 1 = visceral leishmaniasis patients, 2 = malaria patients, 3 = visceral leishmaniasis-malaria co-infected patients.

The Kruskall-Wallis test was used to calculate the *P* values, except for the variable sex for which the Chi-Square test was used, and for the variables DAT titer and *P.f*. parasitaemia, for which the Mann-Whitney test was used.

^†^*P* value refers to differences between groups 1 and 3 only.

^‡^*P* value refers to differences between groups 2 and 3 only.

Sex data based on *a* 28 patients *b* 20 patients *c* 63 patients. Age data based on *d* 27 patients and *e* 63 patients. WBC count data based on *f* 18 patients, *g* 11 patients, *h* 13 patients and *i* 42 patients. *P. falciparum* parasitaemia data based on *j* 12 patients, *k* 10 patients and *l* 22 patients. Two co-infected patients received artemether i.m. three weeks prior to diagnosis and their data on *P. falciparum* parasitaemia were excluded to reduce possible bias.

### Circulating cytokine profiles in VL and malaria mono-infected patients

**Figure 1:**
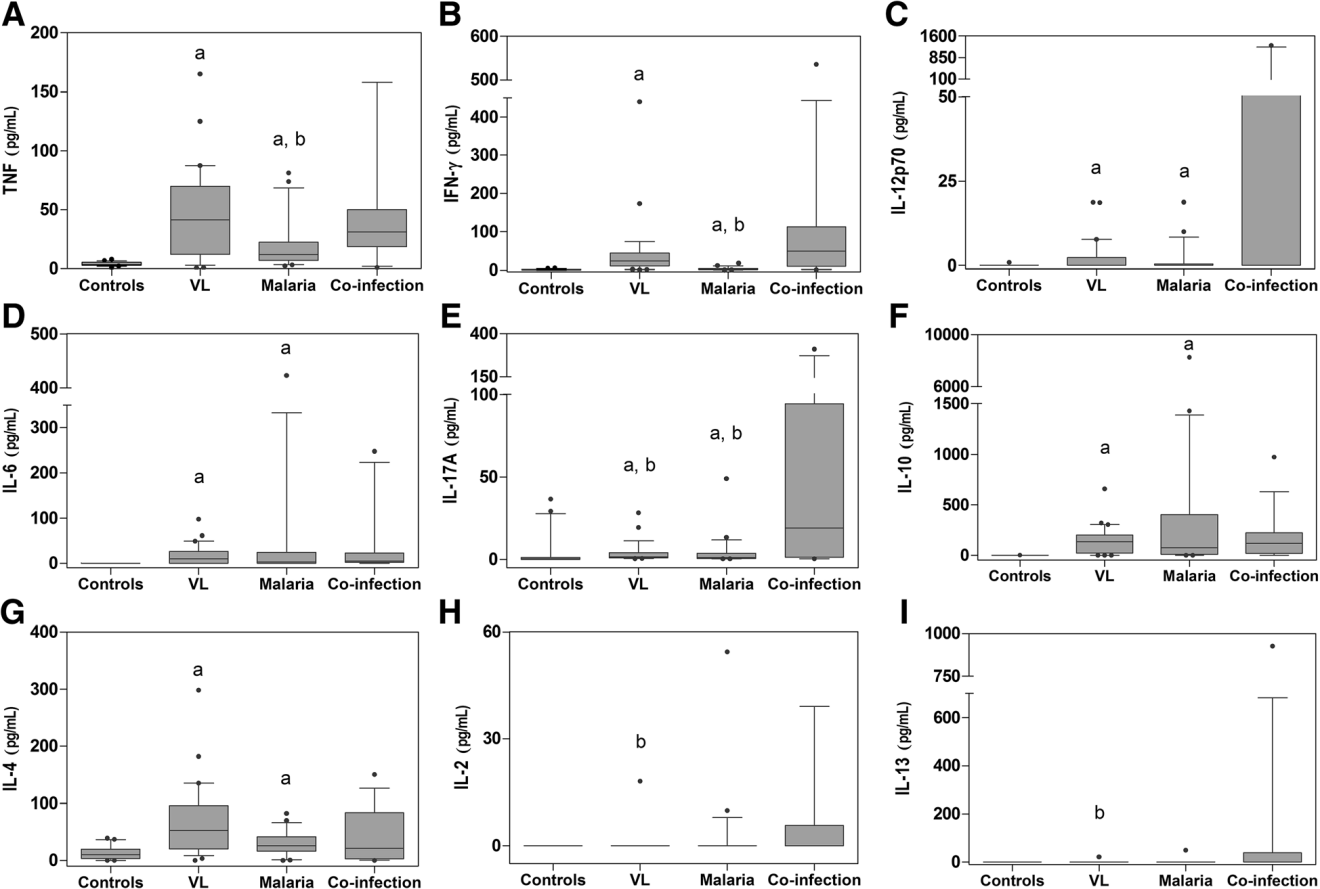
**(A-I) Cytokines from healthy individuals (Controls) and patients with VL and/or malaria.** Cytokine levels in each group (see Additional file 1 for detailed data) are shown as median and interquartile range (box), with 10^th^ and 90^th^ percentiles (whiskers). Letters (a) and (b) above the box-plot indicate statistically significant differences (*P* <0.05) as compared with the healthy controls and the co-infected patients, respectively.

Significantly increased levels of pro-inflammatory cytokines characterized the VL group compared with the healthy individuals (circulating cytokine levels of all study participants are reported in Additional file 1). Tumor necrosis factor, a marker of local and systemic inflammation, and the type-1-inducing cytokine IFN-γ were both strongly up-regulated amongst VL patients, as compared with healthy controls (*P* <0.0001) (Figure 1A and B). Circulating IL-12p70 was also enhanced by the *Leishmania* infection (*P* <0.001): undetectable in all, but one control, this interleukin reached or exceeded the detection limit (0.74 pg/mL) in nearly half of the VL patients (Figure 1C). Similarly, the serum level of IL-6 was negligible in all healthy individuals (<0.97 pg/mL), but raised to a 10-fold higher value in the VL group (*P* <0.0001) (Figure 1D). Systemic inflammation amongst VL patients was confirmed by IL-17A, whose circulating levels appeared to be significantly induced (*P* <0.001) (Figure 1E). Visceral leishmaniasis cases also exhibited an increase in their anti-inflammatory and regulatory cytokine patterns, as shown by the higher amounts of IL-4, and particularly IL-10 (*P* <0.0001) (Figure 1F and G). No difference in the circulating level of IL-2 and IL-13, on the other hand, distinguished VL-affected and healthy individuals, for whom the level of these two cytokines resulted mostly under the assay detection limits (0.54 pg/mL and 0.57 pg/mL, respectively) (Figure 1H and I).

Clinical malaria cases exhibited an immunological profile qualitatively similar to that of the VL patients, with increased concentrations of TNF, IL-6 and IL-10 (*P* <0.0001) and, to a less extent, IFN-γ and IL-17A (*P* <0.01), IL-4 and IL-12p70 (*P* <0.05) (Figure 1A-G). Interleukin-2 and IL-13 persisted at negligible levels (Figure 1H and I).

### Circulating cytokine profiles in VL and malaria co-infected patients

Co-occurrence of malaria and VL in the same patients deeply altered their immunological response as compared with the single infections. Overall, co-infection of *Leishmania* and *Plasmodium* resulted in a marked elevation of type-1 and pro-inflammatory cytokine patterns, presumably triggered by the *Leishmania* disease. Whereas comparable amounts of IFN-γ and TNF were detected in the sera of VL mono- and co-infected patients, the level of these two cytokines significantly raised when malaria patients were co-diagnosed with VL (*P* <0.001 and *P* <0.01, respectively) (Figure 1A and B). Up-regulation of pro-inflammatory IL-17A distinguished the co-infected patients from both VL and malaria mono-infection cases (*P* <0.05) (Figure 1E), indicating that synergistic interactions of the two diseases upon each other concurred to its release. Strikingly different IL-17A concentrations were measured amongst the co-infected patients, whose demographic and clinical characteristics, however, did not display any correlation with the cytokine level. In the co-infected cohort, IL-2 and IL-13 concentrations exceeded the detection limit with a higher frequency (4 out of 15 samples) than amongst VL patients (1 out of 29 samples), resulting in significant differences (*P* <0.05) between the two groups (Figure 1H and I).

**Figure 2:**
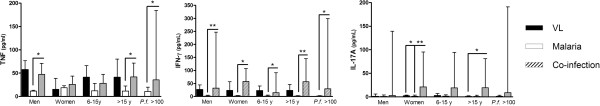
**Cytokines from patients with VL and/or malaria matched by sex, age and *P. falciparum *parasitaemia.** Cytokine levels in each group are shown as median and interquartile range. *P* <0.05*; *P* <0.01**. 6-15y = age 6-15 years; >15y = age >15 years; *P.f*. >100 = *P. falciparum* parasitaemia >100 parasites/μL.

To exclude that the cytokine profiles observed for VL and/or malaria infected patients may have been biased by differences in their demographic and clinical features, the study population was stratified by sex, age or *P. falciparum* parasitaemia (Table 2). For age, patients were matched by means of two groups (6-15 years, >15 years) selected to yield similar group-wise compositions, while for *P. falciparum* parasitaemia, an infection intensity >100 parasites/μL of blood was chosen (corresponding to >0.002%, the level above which patients may become symptomatic). None of the three variables displayed a systematic confounding effect on the cytokines examined here, whose variations amongst the three study groups remained overall stable (Figure 2). Increased concentrations of IFN-γ distinguished the co-infected from the malaria mono-infected patients in all of the matched groups, whereas significant differences in TNF levels were observed for men only and for patients above 15 years of age. Importantly, for TNF and IFN-γ the lower *P. falciparum* infection intensity recorded amongst the co-infected cohort did not flaw the comparison with the malaria patients, as statistically significant differences still distinguished the two groups after parasitaemia stratification. Increase in co-infected patient IL-17A level appeared to be mainly triggered by malaria, as the difference with this group reduced after age and parasitaemia matching, but persisted towards VL patients in women only and in subjects older than 15 years. No significant differences were observed for the remaining six cytokines, including IL-2 and IL-13, for which most patients displayed negligible levels (data not shown).

**Table 2:** **Characteristics of patients with VL and**/**or malaria matched by sex**, **age or *P. falciparum *** **parasitaemia**

**Characteristics**	**Patient group**			
	**1**	**2**	**3**	**Total**
**Subjects ( *n *)**	29	21	15	65
**Male/Female ( *n *)**	20/8	11/9	9/6	40/23
**Age 6-15 years (*n***)	13	14	7	34
Median (Interquartile range)	9 (7-13)	8 (7-9)	9 (7-10)	
**Age >15 years ( *n *)**	14	7	8	29
Median (Interquartile Range)	26 (20-35)	27 (25-30)	19 (17-27)	
***P.f*. parasitaemia** **>100 parasites/μL ( *n *)**	NA	12	4	16
Median (Interquartile range)	NA	825 (355-2325)	640 (290-1088)	

*P.f*. = *Plasmodium falciparum*; *NA* = not applicable.

Groups 1 to 3 are: 1 = visceral leishmaniasis patients, 2 = malaria patients, 3 = visceral leishmaniasis-malaria co-infected patients.

### Correlation between circulating cytokines in VL and malaria co-infected patients

Specific mechanisms cross-regulate the production of cytokines, with positive and negative feed-back loops to control secretion of signaling molecules. To understand the link interconnecting each of the cytokines examined here, their correlation was investigated using the Spearman’s correlation rank test. As shown in Table 3, multiple positive correlations were identified between pro-inflammatory (TNF *vs*. IL-6) and type-1 cytokines (IFN-γ *vs*. IL-12p70), as well as for type-1 *vs.* pro-inflammatory (IFN-γ & IL-12p70 *vs*. TNF, IL-6 & IL-17A), and type-2 *vs.* pro-inflammatory (IL-13 *vs*. TNF, IL-6 & IL-17A) and type-1 cytokines (IL-13 *vs*. IL-12p70 & IFN-γ). Conversely, no correlation could be identified between the examined cytokines and the DAT titers as well as the *P. falciparum* parasitaemia of co-infected patients (data not shown).

**Table 3:** **Correlation coefficients between cytokine levels from VL**-**malaria co**-**infected patients recruited at Tabarak Allah Hospital**, **Sudan**

**Cytokine correlation**									
**Coefficient r**_**s**_^**†**^	**TNF**	**IL-6**	**IL-17A**	**IFN-γ**	**IL-12p70**	**IL-13**	**IL-10**	**IL-4**	**IL-2**
**TNF**		0.80^***^	0.63^*^	0.69^**^	0.66^**^	0.75^**^	0.59^*^	0.40	0.20
**IL-6**			0.55^*^	0.68^**^	0.82^***^	0.68^**^	0.61^*^	0.27	0.13
**IL-17A**				0.64^**^	0.68^**^	0.70^**^	0.14	0.30	0.20
**IFN-γ**					0.75^**^	0.78^**^	0.46	0.59^*^	0.39
**IL-12p70**						0.85^***^	0.34	0.19	0.34
**IL-13**							0.28	0.32	0.53^*^
**IL-10**								0.14	0.12
**IL-4**									-0.15

^†^Spearman’s (r_s_) rank correlations were computed and statistical significance was considered when *P* <0.001^***^, *P* <0.01^**^ or *P* <0.05^*^.

## Discussion

The ability of *Leishmania* and *Plasmodium* parasites to manipulate host immunity and co-inhabit part of the same lymphoid tissues suggests the possibility that the two diseases may interact with each other, when co-occurring in the same host. This is demonstrated for the first time in naturally co-infected patients by the pilot study presented here. Comparative analysis of cytokine profiles from co- and mono-infected patients highlighted substantial variations in the immune response mounted upon co-infection, confirming the ability of *L. donovani* and *P. falciparum* to mutually interact at the immunological level. Patients harboring both leishmanial and malarial parasites responded with an overall increase in type-1 and pro-inflammatory cytokine release, which partly reflected the effect elicited by VL (TNF, IFN-γ) and malaria (IL-2), and partly resulted from a synergistic interaction of the two diseases upon each other (IL-17A). Secretion of IL-13 in co-infected patients significantly exceeded the amounts found in VL patients and displayed positive correlations with most of the examined cytokines. Although this trend can be seen as an attempt of the immune system to contain the effects elicited by type-1 and pro-inflammatory cytokines (these patients exhibited some of the highest concentrations of IFN-γ, TNF and IL-17A), the finding remains poorly representative, reflecting the response of 4 patients only, with the remaining co-infected patients (*n* = 11) displaying negligible levels of IL-13 just as most of VL and malaria patients.

Measurement of IL-17A levels allowed to distinguish the co-infected patients from both VL and malaria mono-infected counterparts, indicating that both diseases synergistically concurred to its up-regulation. Better known for its pro-inflammatory effects in allergic and autoimmune conditions, IL-17 has been recently implicated in the protective immunity towards bacterial, fungal and protozoan infections [55], where it is thought to mediate recruitment of neutrophils to the epithelial and mucosal surfaces and induce production of antimicrobial peptides [55,56]. Its release by CD4^+^ Th17 cells has been associated with resistance to human VL [57] and positive resolution of murine *L. donovani* infections [58], suggesting that Th17 and Th1 cytokines may play complementary roles in parasite clearance. Hence, the increased concentrations of IL-17A found in the co-infected *vs.* the VL mono-infected cohort, besides the already elevated IFN-γ and IL-12p70, may be indicative of a favorable, possibly improved, prognosis for VL, though the present data do not allow to draw conclusions in this respect. In support of this speculation is the finding of a recent study conducted in Barbar el Fugarra, a Sudanese village situated only a few tens of kilometers away from Tabarak Allah Hospital (where patients in this study were recruited), in which peripheral blood mononuclear cells (PBMCs) isolated from VL-seropositive individuals who did not develop disease at any time during the 6-year survey, secreted significantly higher IL-17 levels when challenged with *L. donovani* in comparison with VL-seropositive individuals who became symptomatic within 6 months from the evaluation [57]. Interestingly, malaria appeared as the major trigger of this IL-17A up-regulation in co-infected patients, given that no relationship between patients’ demographic and clinical variables and the corresponding IL-17A serum level could be identified in this group (nor in any other group). Expansion of IL-17-producing cells (either CD4^+^ T cells, CD8^+^ T cells or macrophages) and related cytokines (IL-17, IL-22 and IL-23) has been observed in *P. vivax* natural infections [59] as well as *P. berghei*, *P. chabaudi and P. fragile* animal models [60-62], where these interleukins have been shown to reduce parasite intensity and protect against fatal outcomes [59-61]. Conversely, a clear role of IL-17 immunity in *P. falciparum* infections is yet to be demonstrated. Transcriptional profiling of PBMCs isolated from *P. falciparum*-infected patients has recently highlighted a Th17/Thαβ driven bias in the immune response mounted against malaria, with up-regulation of several Th17- and neutrophil-related genes [63,64] and induction of a NK cell-mediated humoral response *via* interferon α and β [63]. Triggering of this Thαβ immunity, in particular, was shown to inhibit the IL-12 driven Th1 response [63], necessary for boosting clearance of malaria parasites [29,65-67], particularly during the pre-erythocytic stage, when cell-mediated immunity is essential for control of infection. If induction of a Th17 response may, therefore, indirectly impair host ability to contain malaria through suppression of macrophage activities, the IFN-γ dominant response elicited by VL may partially compensate for this deficiency and act as a pre-priming stimulus upon *Plasmodium* infection, for the development of malaria adaptive immunity (*via* NKT cells, e.g.) and the nitric oxygen-dependent suppression of intra-hepatocytic forms. The above-shown data confirm the leading presence of IFN-γ (*P* <0.0001), followed by TNF (*P* <0.05) and IL-4 (*P* <0.05), in the sera of VL patients as compared with the malaria ones, and clearly identify a shift towards type-1/pro-inflammatory polarization when malaria co-occurred with VL. In addition, a significantly reduced *P. falciparum* infection intensity was observed among co-infected patients, suggesting improved tolerance of these individuals to the malaria disease. Whether this reduced susceptibility resulted from the VL-driven pre-immune response remains to be demonstrated. The pioneer work of Adler *et al*. on co-infected hamsters [49] highlighted a reduced proliferation of *P. berghei* for effect of the *Leishmania* infection, supporting the idea of a VL-triggered cross-immunity against malaria, whereas the more recent mouse model data [46-48] seem to suggest the opposite conclusion. It is worthy to note that animals were challenged with blood-stage parasites rather than with sporozoites, bypassing the naturally occurring liver phase against which cellular immunity is most effective and most likely to be developed in response to VL (*Leishmania* parasites visceralize in the liver, too). Moreover, mice and hamsters are not equally representative models of the VL disease, whose clinico-pathological features in humans are better reproduced by the golden hamster model [68].

The exploratory nature of this survey implies its design and findings are limited by the small sample sizes and the lack of subject matching between groups, although no significant difference in the distribution of patients’ demographic and clinical features was observed. Diagnosis of VL in clinical suspects was confirmed by serology, according to the national policy, precluding any analysis on parasite loads and their link with cytokine profiles. Assessment of malaria parasitaemia, on the contrary, was performed on peripheral blood films, but the low sensitivity of microscopy observation inevitably limits its reliability as a quantitative assay. Moreover, in the absence of a molecular screening of the recruited individuals, the risk of sub-microscopy malaria infections being carried by the VL patients and/or apparently healthy controls cannot be excluded. Malaria mono- and co-infected patients exhibited different *P. falciparum* blood parasitaemias. Whether these differences are linked to their particular diagnosis, however, is unknown, as patients were recruited sequentially and discernment between clinical and sub-clinical co-infection cases is not possible if one of the two diseases manifests with symptoms. Therefore, recruitment of asymptomatic, but parasitaemic individuals for each of the two infections may be useful to control for non-homogeneous group-wise comparisons. Absence of pre-existent disorders was based on patient reporting only, with no diagnostic procedure performed, other than those ones aimed to confirm VL or malaria. Finally, longitudinal rather than cross-sectional assessments should be endorsed, as they could help identifying those fundamental associations amongst parasite load, cytokine response and clinical picture which are keys to the interpretation of present data. Similar studies may not only clarify the exact role of the VL-malaria co-infection on *P. falciparum* proliferation, but they would be pivotal for understanding the clinical implications that arise from the different cytokine profiles.

## Conclusions

Immune-mediated interactions between *L. donovani* complex and *P. falciparum* appear to shape the immunological response taking place in the co-infected host and possibly the intensity of infections that follow. Similar scenarios have been depicted with other malaria co-infections, indicating that the potential implications arising from multiple pathogen-host relations should be addressed when designing malaria vaccine trials. Careful consideration of parasite interplays should be taken when defining the best strategy for clinical management of VL-malaria co-infections, to ensure that immune homeostasis may be restored without harming patient’s clinical course.

## Abbreviations

VL: Visceral leishmaniasis; IFN-γ: Interferon-gamma; IL: Interleukin; TNF: Tumor necrosis factor; Th: T helper; NK: Natural killer; TGF-β: Transforming growth factor-beta; WBC: White blood cell; WHO: World Health Organization; DAT: Direct agglutination test; MΦ: Macrophage; Hb: Hemoglobin; P.f: *Plasmodium falciparum*; PBMC: Peripheral blood mononuclear cell.

## Competing interests

The authors declare that they have no competing interests.

## Authors’ contribution

EvdB designed the study, carried out the cytokine measurement and drafted the manuscript. AT recruited the study participants, performed collection of data and samples and participated to the sample analysis. MS performed the statistical analysis of the data. PM and EM conceived of the study, participated in its design, contributed to the sample analysis and helped to draft the manuscript. MG revised the critical content of the manuscript. BN participated to the study design and coordinated the work in the field. HS conceived of the study, participated in its design and coordination, contributed to the sample analysis and helped to draft the manuscript. All authors read and approved the final manuscript.

## Supplementary Material

Additional file 1 Cytokine levels (pg/mL) as measured in all patient samples. Click here for file

## References

[B1] Cattadori IM Boag B Hudson PJ Parasite co-infection and interaction as drivers of host heterogeneity Int J Parasitol 2008 38 3–4 371 380 17936286 10.1016/j.ijpara.2007.08.004

[B2] Cattadori IM Albert R Boag B Variation in host susceptibility and infectiousness generated by co-infection: the myxoma-*Trichostrongylus retortaeformis* case in wild rabbits J R Soc Interface 2007 4 16 831 840 10.1098/rsif.2007.1075 17580288 PMC2386892

[B3] Ezenwa VO Jolles AE From host immunity to pathogen invasion: the effects of helminth coinfection on the dynamics of microparasites Integr Comp Biol 2011 51 4 540 551 10.1093/icb/icr058 21727178

[B4] Cox FEG Concomitant infections, parasites and immune responses Parasitology 2001 122 Suppl 122 S23 S38 11442193 10.1017/s003118200001698x

[B5] Supali T Verweij JJ Wiria AE Djuardi Y Hamid F Kaisar MM Wammes LJ van Lieshout L Luty AJ Sartono E Yazdanbakhsh M Polyparasitism and its impact on the immune system Int J Parasitol 2010 40 10 1171 1176 10.1016/j.ijpara.2010.05.003 20580905

[B6] Graham AL Cattadori IM Lloyd-Smith JO Ferrari MJ Bjørnstad ON Transmission consequences of coinfection: cytokines writ large? Trends Parasitol 2007 23 6 284 291 10.1016/j.pt.2007.04.005 17466597

[B7] Page KR Scott AL Manabe YC The expanding realm of heterologous immunity: friend or foe? Cell Microbiol 2006 8 2 185 196 10.1111/j.1462-5822.2005.00653.x 16441430

[B8] Kourilsky P Truffa-Bachi P Cytokine fields and the polarization of the immune response Trends Immunol 2001 22 9 502 509 10.1016/S1471-4906(01)02012-9 11525941

[B9] van den Bogaart E Berkhout MM Adams ER Mens PF Sentongo E Mbulamberi DB Straetemans M Schallig HD Chappuis F Prevalence, features and risk factors of malaria co-infections among visceral leishmaniasis patients from Amudat Hospital Uganda PLoS Negl Trop Dis 2012 6 4 e1617 10.1371/journal.pntd.0001617 PMC3323524 22506087

[B10] van den Bogaart E Berkhout MM Nour AB Mens PF Talha AB Adams ER Ahmed HB Abdelrahman SH Ritmeijer K Nour BY Schallig HD Concomitant malaria among visceral leishmaniasis in-patients from Gedarif and Sennar States, Sudan: a retrospective case-control study BMC Public Health 2013 13 332 10.1186/1471-2458-13-332 23577673 PMC3659061

[B11] Stäger S Joshi T Bankoti R Immune evasive mechanisms contributing to persistent *Leishmania donovani* infection Immunol Res 2010 47 1–3 14 24 20087685 10.1007/s12026-009-8135-4

[B12] Olivier M Gregory DJ Forget G Subversion mechanisms by which *Leishmania* parasites can escape the host immune response: a signaling point of view Clin Microbiol Rev 2005 18 2 293 305 10.1128/CMR.18.2.293-305.2005 15831826 PMC1082797

[B13] Bacellar O Brodskyn C Carvalho EM Barral-Netto M Costa CH Coffman RL Johnson WD Carvalho EM Interleukin-12 restores interferon-γ production and cytotoxic responses in visceral leishmaniasis J Infect Dis 1996 173 6 1515 1518 10.1093/infdis/173.6.1515 8648233

[B14] Hailu A van Baarle D Knol GJ Berhe N Miedema F Kager PA T cell subset and cytokine profiles in human visceral leishmaniasis during active and asymptomatic or sub-clinical infection with *Leishmania donovani* Clin Immunol 2005 117 2 182 191 10.1016/j.clim.2005.06.015 16125466

[B15] Karp CL El-Safi SH Wynn TA Satti MM Kordofani AM Hashim FA Hag-Ali M Neva FA Nutman TB Sacks DL In vivo cytokine profiles in patients with kala-azar: marked elevation of both interleukin-10 and interferon-gamma J Clin Invest 1993 91 4 1644 1648 10.1172/JCI116372 8097208 PMC288142

[B16] Nylén S Maurya R Eidsmo L Manandhar KD Sundar S Sacks D Splenic accumulation of IL-10 mRNA in T cells distinct from CD4 + CD25+ (Foxp3) regulatory T cells in human visceral leishmaniasis J Exp Med 2007 204 4 805 817 10.1084/jem.20061141 17389235 PMC2118563

[B17] Ansari NA Saluja S Salotra P Elevated levels of interferon-γ, interleukin-10, and interleukin-6 during active disease in Indian kala azar Clin Immunol 2006 119 3 339 345 10.1016/j.clim.2006.01.017 16540374

[B18] Nylén S Sacks D Interleukin-10 and the pathogenesis of human visceral leishmaniasis Trends Immunol 2007 28 9 378 384 10.1016/j.it.2007.07.004 17689290

[B19] McGuirk P Mills KHG Pathogen-specific regulatory T cells provoke a shift in the Th1/Th2 paradigm in immunity to infectious diseases Trends in Immunol 2002 23 9 450 455 10.1016/S1471-4906(02)02288-3 12200067

[B20] Ghalib HW Piuvezam MR Skeiky YAW Siddig M Hashim FA El-Hassan AM Russo DM Reed SG Interleukin 10 production correlates with pathology in human *Leishmania donovani* infections J Clin Invest 1993 92 1 324 329 10.1172/JCI116570 8326000 PMC293600

[B21] Walther M Tongren JE Andrews L Korbel D King E Fletcher H Andersen RF Bejon P Thompson F Dunachie SJ Edele F de Souza JB Sinden RE Gilbert SC Riley EM Hill AV Upregulation of TGF-beta, FOXP3, and CD4^+^CD25^+^ regulatory T cells correlates with more rapid parasite growth in human malaria infection Immunity 2005 23 3 287 296 10.1016/j.immuni.2005.08.006 16169501

[B22] Todryk SM Bejon P Mwangi T Plebanski M Urban B Marsh K Hill AV Flanagan KL Correlation of memory T cell responses against TRAP with protection from clinical malaria, and CD4 CD25 high T cells with susceptibility in Kenyans PLoS ONE 2008 3 e2027 10.1371/journal.pone.0002027 18446217 PMC2323567

[B23] Torcia MG Santarlasci V Cosmi L Clemente A Maggi L Mangano VD Verra F Bancone G Nebie I Sirima BS Liotta F Frosali F Angeli R Severini C Sannella AR Bonini P Lucibello M Maggi E Garaci E Coluzzi M Cozzolino F Annunziato F Romagnani S Modiano D Functional deficit of T regulatory cells in Fulani, an ethnic group with low susceptibility to *Plasmodium falciparum* malaria Proc Natl Acad Sci U S A 2008 105 2 646 651 10.1073/pnas.0709969105 18174328 PMC2206590

[B24] Walther M Jeffries D Finney OC Njie M Ebonyi A Deininger S Lawrence E Ngwa-Amambua A Jayasooriya S Cheeseman IH Gomez-Escobar N Okebe J Conway DJ Riley EM Distinct roles for FOXP3 and FOXP3 CD4 T cells in regulating cellular immunity to uncomplicated and severe *Plasmodium falciparum* malaria PLoS Pathog 2009 5 4 e1000364 10.1371/journal.ppat.1000364 19343213 PMC2658808

[B25] Minigo G Woodberry T Piera KA Salwati E Tjitra E Kenangalem E Price RN Engwerda CR Anstey NM Plebanski M Parasite-dependent expansion of TNF receptor II-positive regulatory T cells with enhanced suppressive activity in adults with severe malaria PLoS Pathog 2009 5 4 e1000402 10.1371/journal.ppat.1000402 19390618 PMC2668192

[B26] Ferreira A Schofield L Enea V Schellekens H Van der Meide P Collins WE Nussenzweig RS Nussenzweig V Inhibition of development of exoerythrocytic forms of malaria parasites by gamma-interferon Science 1986 232 4752 881 10.1126/science.3085218 3085218

[B27] Ferreira A Enea V Morimoto T Nussenzweig V Interferon-gamma inhibits the intrahepatocytic development of malaria parasites *in vitro* J Immunol 1987 139 6 2020 2025 2957445

[B28] McCall MB Sauerwein RW Interferon-γ–central mediator of protective immune responses against the pre-erythrocytic and blood stage of malaria J Leukoc Biol 2010 88 6 1131 1143 10.1189/jlb.0310137 20610802

[B29] Lumsden JM Schwenk RJ Rein LE Protective immunity induced with the RTS, S/AS vaccine is associated with IL-2 and TNF-alpha producing effector and central memory CD4 T cells PLoS ONE 2011 6 7 e20775 10.1371/journal.pone.0020775 21779319 PMC3136919

[B30] Nussler A Pied S Goma J Renia L Miltgen F Grau GE Mazier D TNF inhibits malaria hepatic stages *in vitro* via synthesis of IL-6 Int Immunol 1991 3 4 317 321 10.1093/intimm/3.4.317 1878339

[B31] Sun P Schwenk R White K Stoute JA Cohen J Ballou WR Voss G Kester KE Heppner DG Krzych U Protective immunity induced with malaria vaccine, RTS, S, is linked to Plasmodium falciparum circumsporozoite protein-specific CD4+ and CD8+ T cells producing IFN-gamma J Immunol 2003 171 12 6961 6967 10.4049/jimmunol.171.12.6961 14662904

[B32] Cabantous S Poudiougou B Traore A Keita M Cisse MB Doumbo O Dessein AJ Marquet S Evidence that interferon-gamma plays a protective role during cerebral malaria J Infect Dis 2005 192 5 854 860 10.1086/432484 16088835

[B33] D’Ombrain MC Robinson LJ Stanisic DI Taraika J Bernard N Michon P Mueller I Schofield L Association of early interferon-gamma production with immunity to clinical malaria: a longitudinal study among Papua New Guinean children Clin Infect Dis 2008 47 11 1380 1387 10.1086/592971 18947328

[B34] Robinson LJ D’Ombrain MC Stanisic DI Taraika J Bernard N Richards JS Beeson JG Tavul L Michon P Mueller I Schofield L Cellular tumor necrosis factor, gamma interferon, and interleukin-6 responses as correlates of immunity and risk of clinical *Plasmodium falciparum* malaria in children from Papua New Guinea Infect Immun 2009 77 7 3033 3043 10.1128/IAI.00211-09 19380468 PMC2708537

[B35] Luty AJ Lell B Schmidt-Ott R Luckner D Greve B Matousek P Herbich K Schmid D Migot-Nabias F Deloron P Nussenzweig RS Kremsner PG Interferon-gamma responses are associated with resistance to reinfection with *Plasmodium falciparum* in young African children J Infect Dis 1999 179 4 980 988 10.1086/314689 10068595

[B36] Roestenberg M McCall M Hopman J Wiersma J Luty AJ van Gemert GJ van de Vegte-Bolmer M van Schaijk B Teelen K Arens T Spaarman L de Mast Q Roeffen W Snounou G Rénia L van der Ven A Hermsen CC Sauerwein R Protection against a malaria challenge by sporozoite inoculation N Engl J Med 2009 361 5 468 477 10.1056/NEJMoa0805832 19641203

[B37] Doolan DL Hoffman SL The complexity of protective immunity against liver-stage malaria J Immunol 2000 165 3 1453 1462 10.4049/jimmunol.165.3.1453 10903750

[B38] Perlaza BL Sauzet JP Brahimi K BenMohamed L Druilhe P Interferon-gamma, a valuable surrogate marker of *Plasmodium falciparum* pre-erythrocytic stages protective immunity Malar J 2011 10 1 27 10.1186/1475-2875-10-27 21303495 PMC3046914

[B39] Artavanis-Tsakonas K Riley EM Innate immune response to malaria: rapid induction of INF-gamma from human NK cells by live *Plasmodium falciparum*-infected erythrocytes J Immunol 2002 169 6 2956 2963 10.4049/jimmunol.169.6.2956 12218109

[B40] Teirlinck AC McCall MBB Roestenberg M Scholzen A Woestenenk R de Mast Q van der Ven AJ Hermsen CC Luty AJ Sauerwein RW Longevity and composition of cellular immune responses following experimental *Plasmodium falciparum* malaria infection in humans PLoS Pathog 2011 7 12 e1002389 10.1371/journal.ppat.1002389 22144890 PMC3228790

[B41] Hensmann M Kwiatkowski D Cellular basis of early cytokine response to *Plasmodium falciparum* Infect Immun 2001 69 4 2364 2371 10.1128/IAI.69.4.2364-2371.2001 11254594 PMC98166

[B42] Biemba G Gordeuk VR Thuma P Weiss G Markers of inflammation in children with several malarial anaemia Trop Med Int Health 2000 5 4 256 262 10.1046/j.1365-3156.2000.00545.x 10810019

[B43] Rovira-Vallbona E Moncunill G Bassat Q Aguilar R Machevo S Puyol L Quintó L Menéndez C Chitnis CE Alonso PL Dobaño C Mayor A Low antibodies against *Plasmodium falciparum* and imbalanced pro-inflammatory cytokines are associated with severe malaria in Mozambican children: a case-control study Malar J 2012 11 181 10.1186/1475-2875-11-181 22646809 PMC3464173

[B44] Perkins DJ Weinberg JB Kremsner PG Reduced interleukin-12 and transforming growth factor-beta1 in severe childhood malaria: relationship of cytokine balance with disease severity J Infect Dis 2000 182 3 988 992 10.1086/315762 10950804

[B45] Hunt NH Grau GE Cytokines: accelerators and brakes in the pathogenesis of cerebral malaria Trends Immunol 2003 24 9 491 499 10.1016/S1471-4906(03)00229-1 12967673

[B46] Marques CS Rolão N Centeno-Lima S Lousada H Maia C Campino L do Rosário VE Silveira H Studies in a co-infection murine model of *Plasmodium chabaudi chabaudi* and *Leishmania infantum*: interferon-gamma and interleukin-4 mRNA expression Mem Inst Oswaldo Cruz 2005 100 8 889 892 10.1590/S0074-02762005000800011 16444421

[B47] Coleman RE Edman JD Semprevivo LH *Leishmania mexicana*: effect of concomitant malaria on cutaneous leishmaniasis. Development of lesions in a *Leishmania*-susceptible (BALB/c) strain of mouse Exp Parasitol 1988 65 2 269 276 10.1016/0014-4894(88)90131-2 3350106

[B48] Coleman RE Edman JD Semprevivo LH Interactions between *Plasmodium yoelii* and *Leishmania mexicana amazonensis* in Leishmania resistant C57B1/6 mice Am J Trop Med Hyg 1988 39 6 540 544 3207174 10.4269/ajtmh.1988.39.540

[B49] Adler S The behaviour of *Plasmodium berghei* in the golden hamster *Mesocricetus auratus* infected with visceral leishmaniasis Trans R Soc Trop Med Hyg 1954 48 5 431 440 10.1016/0035-9203(54)90145-5 13216948

[B50] Center for Disease Control and Prevention Guidelines for the Use of Antiretroviral Agents in Pediatric HIV Infection 1998 Atlanta: MMWR 47(No. RR-4) 9572665

[B51] Comans-Bitter WM de Groot R van den Beemd R Neijens HJ Hop WC Groeneveld K Hooijkaas H van Dongen JJ Immunophenotyping of blood lymphocytes in childhood. Reference values for lymphocyte subpopulations J Pediatr 1997 130 3 388 393 10.1016/S0022-3476(97)70200-2 9063413

[B52] Warhurst DC Williams JE Laboratory diagnosis of malaria J Clin Pathol 1996 49 7 533 538 10.1136/jcp.49.7.533 8813948 PMC500564

[B53] Harith AE Kolk AH Kager PA Leeuwenburg J Muigai R Kiugu S Laarman JJ A simple and economical direct agglutination test for serodiagnosis and seroepidemiological studies of visceral leishmaniasis Trans R Soc Trop Med Hyg 1986 80 4 583 587 10.1016/0035-9203(86)90149-5 3101241

[B54] World Health Organization Expert Committee Control of the Leishmaniases 1990 Geneva: WHO Technical Report Series No. 793 2124015

[B55] O’Connor W Jr Zenewicz LA Flavell RA The dual nature of T (H) 17 cells: shifting the focus to function Nat Immunol 2010 11 6 471 476 10.1038/ni.1882 20485275

[B56] Liang SC Tan XY Luxenberg DP Karim R Dunussi-Joannopoulos K Collins M Fouser LA Interleukin (IL)-22 and IL-17 are coexpressed by Th17 cells and cooperatively enhance expression of antimicrobial peptides J Exp Med 2006 203 10 2271 2279 10.1084/jem.20061308 16982811 PMC2118116

[B57] Pitta MGR Romano A Cabantous S Henri S Hammad A Kouriba B Argiro L el Kheir M Bucheton B Mary C El-Safi SH Dessein A IL-17 and IL-22 are associated with protection against human kala azar caused by *Leishmania donovani* J Clin Invest 2009 119 8 2379 2387 19620772 10.1172/JCI38813 PMC2719936

[B58] Ghosh K Sharma G Saha A Kar S Das PK Ukil A Successful therapy of visceral leishmaniasis with curdlan involves T-helper 17 cytokines J Infect Dis 2013 207 6 1016 1025 10.1093/infdis/jis771 23255562

[B59] Bueno LL Morais CG Lacerda MV Fujiwara RT Braga ÉM Interleukin-17 producing T helper cells are increased during natural Plasmodium vivax infection Acta Trop 2012 123 1 53 57 10.1016/j.actatropica.2012.02.071 22476130

[B60] Ishida H Imai T Suzue K Hirai M Taniguchi T Yoshimura A Iwakura Y Okada H Suzuki T Shimokawa C Hisaeda H IL-23 protection against Plasmodium berghei infection in mice is partially dependent on IL-17 from macrophages Eur J Immunol 2013 43 10 2696 2706 10.1002/eji.201343493 23843079

[B61] Mastelic B Do Rosario AP Veldhoen M Renauld JC Jarra W Spoonas AM Roetynck S Stockinger B Langhorne J IL-22 protects against liver pathology and lethality of an experimental blood-stage malaria infection Front Immunol 2012 3 85 22566965 10.3389/fimmu.2012.00085 PMC3342387

[B62] Ryan-Payseur B Ali Z Huang D Chen CY Yan L Wang RC Collins WE Wang Y Chen ZW Virus infection stages and distinct Th1 or Th17/Th22 T-cell responses in malaria/SHIV coinfection correlate with different outcomes of disease J Infect Dis 2011 204 9 1450 1462 10.1093/infdis/jir549 21921207 PMC3218650

[B63] Hu WC Human immune responses to *Plasmodium falciparum* infection: molecular evidence for a suboptimal THalphabeta and TH17 bias over ideal and effective traditional TH1 immune response Malar J 2013 12 1 392 10.1186/1475-2875-12-392 24188121 PMC3928643

[B64] Griffiths MJ Shafi MJ Popper SJ Hemingway CA Kortok MM Wathen A Rockett KA Mott R Levin M Newton CR Marsh K Relman DA Kwiatkowski DP Genomewide analysis of the host response to malaria in Kenyan children J Infect Dis 2005 191 10 1599 1611 10.1086/429297 15838786

[B65] Doolan DL Martinez-Alier N Immune response to pre-erythocytic stages of malaria parasites Cur Mol Med 2006 6 2 169 185 10.2174/156652406776055249 16515509

[B66] Puri SK Maheshwari RK Dutta GP Friedman RM Dhar MM Human interferon-gamma protects rhesus monkeys against sporozoite-induced *Plasmodium cynomolgi* malaria infection J Interferon Res 1988 8 2 201 206 10.1089/jir.1988.8.201 3132512

[B67] Donovan MJ Messmore AS Scrafford DA Sacks DL Kamhawi S Kamhawi S McDowell MA Uninfected mosquito bites confer protection against infection with malaria parasites Infect Immun 2007 75 5 2523 2530 10.1128/IAI.01928-06 17339356 PMC1865743

[B68] De Oliveira CI Teixeira MJ Gomes R Barral A Brodskyn C Animal models for infectious diseases caused by parasites: Leishmaniasis Drug Dis Today: Dis Mod 2004 1 1 81 86 10.1016/j.ddmod.2004.07.005

